# Pyrrolizidine alkaloids and health risk of three Boraginaceae used in TCM

**DOI:** 10.3389/fphar.2023.1075010

**Published:** 2023-03-23

**Authors:** Ke Zan, Zhao Wang, Xiao-Wen Hu, Yao-Lei Li, Ying Wang, Hong-Yu Jin, Tian-Tian Zuo, Shuang-Cheng Ma

**Affiliations:** ^1^ National Institutes for Food and Drug Control, Beijing, China; ^2^ School of Chinese Materia Medica, Beijing University of Chinese Medicine, Beijing, China

**Keywords:** *Arnebia euchroma*, *Arnebia guttata*, *Lithospermum erythrorhizon*, pyrrolizidine alkaloids, risk assessment, UHPLC-MS/MS, relative potency (REP)

## Abstract

**Objective:** The aim of this study was to systematically explore the pyrrolizidine alkaloids (PAs) type, content and risk assessment in the three Boraginaceae used in TCM, involving Arnebia euchroma (AE), A. guttata (AG), and Lithospermum erythrorhizon (LE).

**Method:** A UHPLC–MS/MS method was established to simultaneously determine eight pyrrolizidine alkaloids (PAs), namely intermedine, lycopsamine, intermedine N-oxide, lycopsamine N-oxide, 7-acetyllycopsamine, 7-acetyllycopsamine N-oxide, echimidine N-oxide, and echimidine in the three herbs. Based on these results, the risk assessment was explored using the routine margin of exposure (MOE) combined with relative potency (REP) for oral and external usage, respectively.

**Results and Conclusion:** Imermedine and imermedine N-oxide were common components in the eight tested PAs. 7-acetyllycopsamine and its N-oxide were not detected in AE; echimidine and its N-oxide were not detected in AG; lycopsamine and its N-oxide, 7-acetyllycopsamine and its N-oxide were not detected in LE. The total contents of 8 PAs in 11 batches of AG was341.56–519.51 μg/g; the content in 15 batches of LE was 71.16–515.73 μg/g, and the content in 11 batches of AE was 23.35–207.13 μg/g. Based on these results, the risk assessment was explored using MOE combined with REP for oral and external usage, respectively. The findings of the risk assessment method of PAs based on MOE combined with the REP factor were consistent with the clinical toxicity results. As an oral herb, AE had low risk or no risk due to its low PA contents, and individual batches of LE were medium risk, while attention should be paid to their clinical use.AG was also low risk. The external use of the three Boraginaceae used in TCM was not associated with any risk. This study systematically explored the PA type and content of the three Boraginaceae used in TCM. Additionally, the refined risk assessment of PAs based on REP provided a more scientific basis for quality evaluation and rational use of the medicinal Boraginaceae used in TCM to improve public health.

## Highlights

- The sensitive UHPLC-MS/MS method was established for analyzing three Boraginaceae used in TCM.
- The contents of pyrrolizidine alkaloids in the three Boraginaceae used in TCM were compared.
- Risk assessment was calculated by routine and relative potency factor methods for oral and external usage.
- The risk results combined with the relative potency factor were consistent with their clinical toxicities.

## 1 Introduction

Pyrrolizidine alkaloids (PAs) are naturally occurring phytotoxins found in about 3% of flowering plants, most of which belong to the families Compositae, Boraginaceae, Orchidaceae, and Leguminosae (Steinhoff, 2019). Currently, there are more than 660 known PAs and their nitrogen oxides. Moreover, 1, 2-unsaturated PAs have been associated with adverse consequences such as hepatotoxicity, nephrotoxicity, genotoxicity, mutagenicity, and carcinogenesis (European Medicines Agency, 2016; Knutsen et al., 2017; Chen et al., 2019). Using a large amount of PAs overa short periodof time can lead to hepatic sinusoidal obstruction syndrome (Zhuge et al., 2019; Teschke et al., 2021). While almost all Boraginaceae plants contain PAs, some are used in traditional Chinese medicine (El-Shazly and Wink, 2014; Ahmad et al., 2018; Stefova et al., 2022). The roots of (Royle ex Benth.) I.M. Johnst. (AE), *Arnebia guttata* Bunge (AG) and *Lithospermum erythrorhizon* Siebold & Zucc. (LE) are three “Zicao” legally approved for use in China (N. P. Committee, 2000; N. P. Committee, 2020), which were used to treat skin diseases such as itching and eczema. In China, there are many oral prescriptions containing Zicao. PAs exist in Zicao, and many studies on the isolation and identification of PAs from Zicao have been reported. Roeder and Rengel (1990) separated and identified imermedine (Im) from LE. Jeong and Lim (2019) established an ultra-high-performance liquid chromatography–quadrupole time-of-flight–mass spectrometry (UHPLC-QTOF-MS) to identify lycopamine (Ly) and lycopamine *N*-oxide (LyN) from the root of LE and determine the content of Ly. Smyrska-Wieleba and colleagues identified Im, imermedine *N*-oxide (ImN), and echimidine *N*-oxide (EmN) from AE by the hydrophilic interaction liquid chromatography–mass spectrometry (HILIC-MS) method (Smyrska-wieleba et al., 2017). However, these reports only detected or determined individual PAs, while there are very few comprehensive analysis reports on PAs in Zicao.

Considering the lack of strong ultraviolet absorption and relatively low content, liquid chromatography–tandem mass spectrometry (LC-MS) is the preferred method for determining PAs due to its high sensitivity and versatility (Luo et al., 2019; Han et al., 2021).

In the pre-experiment, we screened many commercially purchased reference standards by comparing the retention time and fragment ions of ultra-high-performance liquid chromatography coupled with tandem mass spectrometry (UHPLC–MS/MS). Most of the PAs could not be detected in the three Zicao. Besides Im, ImN, Ly, LyN and EmN, echimidine (Em), 7-*O*-acetyllycopsamine (AcLy), and 7-*O*-acetyllycopsamine *N*-oxide (AcLyN) were also detected from at least one Zicao. In this study, a UHPLC–MS/MS method was established to simultaneously determine the above-mentioned eight PAs in Zicao. The toxicity of PAs was closely related to their structures. The risk calculated by the conventional MOE method using the benchmark dose lower confidence limit for 10% (BMDL_10_) value of riddelliine as the reference point for PAs was relatively high as riddelliine is a cyclic di-ester PA belonging to the category with the highest toxicity (Schrenk et al., 2020). According to clinical or animal experiments, Zicao is considered a non-toxic or less toxic medicinal material (Han et al., 2015; Nam et al., 2015; Qian et al., 2021). In their study, Merz and Schrenk (2016) proposed the concept of relative potency (REP) factors based on the toxicity of PAs with different structural types. This study systematically explored the PA type and content of the three medicinal Zicao. Based on the results, the risk assessment was explored by the routine margin of exposure (MOE) combined with REP factors for oral and external usage. The risk assessment was calculated by the conventional MOE method combined with REP, and the results were consistent with the toxicity of Zicao.

## 2 Materials and methods

### 2.1 Plant materials, chemicals, and reagents

**FIGURE 1:**
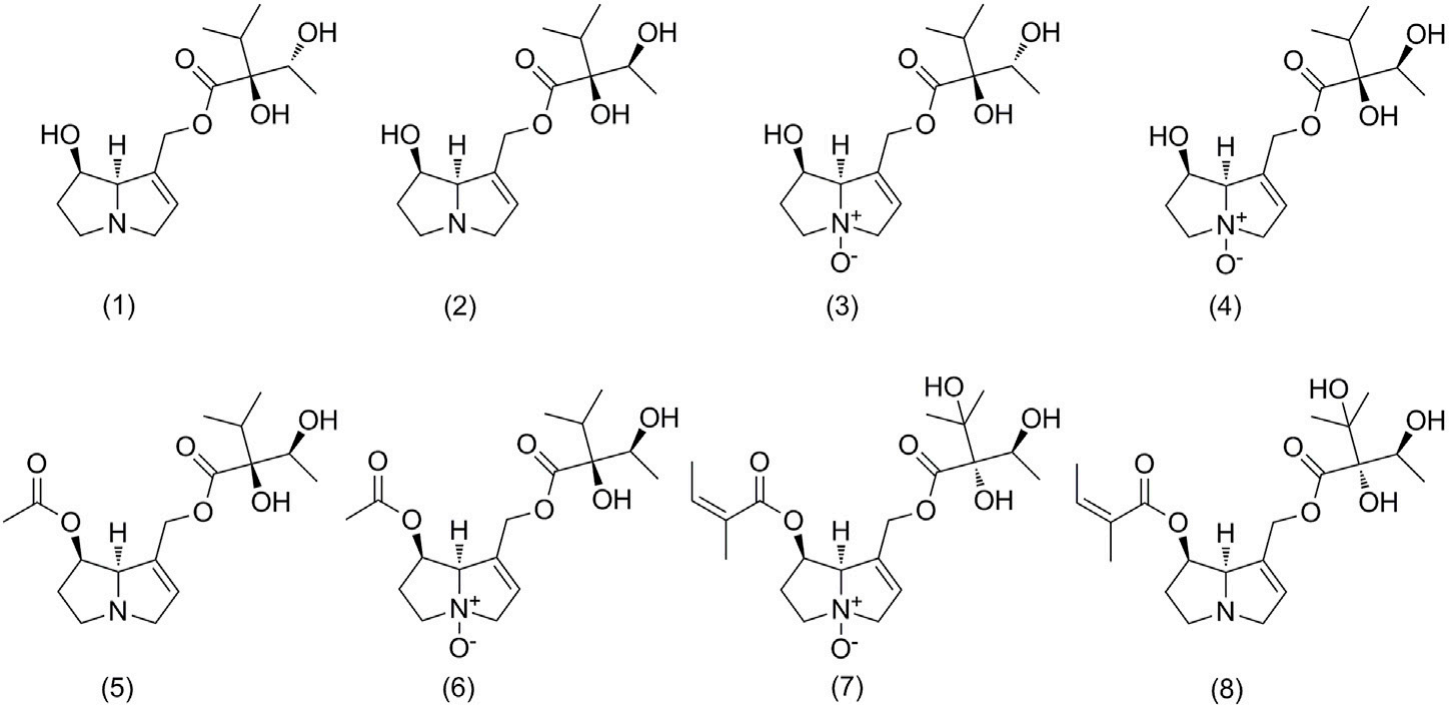
Chemical structures of pyrrolizidine alkaloids analyzed in *Arnebia euchroma*, *A. guttata*, and *Lithospermum erythrorhizon*. Intermedine (1), lycopsamine (2), intermedine *N*-oxide (3), lycopsamine *N*-oxide (4), 7-acetyllycopsamine (5), 7-acetyllycopsamine *N*-oxide (6), echimidine *N*-oxide (7), and echimidine (8).

The following PA standards were obtained from PhytoLab (Vestenbergsgreuth, Germany): Im, Ly, ImN, LyN, AcLy, AcLyN, Em, and EmN. Their structures are presented in Figure 1. Acetonitrile (ACN), formic acid, and methanol were MS grades from Merck (Darmstadt, Germany). Ultra-pure water was prepared using a Millipore Direct Q5 purification system (Millipore, MA, United States). All other used chemicals were of analytical grade and obtained from Sinopharm Chemical Reagent Co., Ltd. (Shanghai, China). All samples were collected from China in September and October 2021, and originally identified by Dr. Ke Zan (National Institutes for Food and Drug Control). The specific place of origin is listed in Table 1. After collection, the herbs were dried at a temperature < 40°C.

**TABLE 1:** Sample information.

Sample code	Species	Parts	Location of collection
AE-1	*A. euchroma*	Root	Bole county, Bortala prefecture, Xinjiang, China
AE-2	*A. euchroma*	Root	Wenquan county, Bortala prefecture, Xinjiang, China
AE-3	*A. euchroma*	Root	Nilka county, Ili prefecture, Xinjiang, China
AE-4	*A. euchroma*	Root	Zhaosu county, Ili prefecture, Xinjiang, China
AE-5	*A. euchroma*	Root	Gongliu county, Ili prefecture, Xinjiang, China
AE-6	*A. euchroma*	Root	Yecheng county, Kashgar prefecture, Xinjiang, China
AE-7	*A. euchroma*	Root	Hejing county, Bayingol prefecture, Xinjiang, China
AE-8	*A. euchroma*	Root	Bugur county, Bayingol prefecture, Xinjiang, China
AE-9	*A. euchroma*	Root	Shawan county, Tarbagatay prefecture, Xinjiang, China
AE-10	*A. euchroma*	Root	Toli county, Tarbagatay prefecture, Xinjiang, China
AE-11	*A. euchroma*	Root	Yumin county, Tarbagatay prefecture, Xinjiang, China
AG-1	*A. guttata*	Root	Urad Front Banner, Bayan Nur, Inner Mongolia, China
AG-2	*A. guttata*	Root	Dengkou county, Bayan Nur, Inner Mongolia, China
AG-3	*A. guttata*	Root	Wuyuan county, Bayan Nur, Inner Mongolia, China
AG-4	*A. guttata*	Root	Alxa left Banner, Alxa League, Inner Mongolia, China
AG-5	*A. guttata*	Root	Ejina Banner, Alxa League, Inner Mongolia, China
AG-6	*A. guttata*	Root	Wuda district, Wuhai, Inner Mongolia, China
AG-7	*A. guttata*	Root	Guyang county, Baotou, Inner Mongolia, China
AG-8	*A. guttata*	Root	Linxi county, Chifeng, Inner Mongolia, China
AG-9	*A. guttata*	Root	Ningcheng county, Chifeng, Inner Mongolia, China
AG-10	*A. guttata*	Root	Minqin county, Wuwei, Gansu, China
AG-11	*A. guttata*	Root	Minqin county, Wuwei, Gansu, China
LE-1	*L. erythrorhizon*	Root	Liaoyang county, Liaoyang, Liaoning, China
LE-2	*L. erythrorhizon*	Root	Dengta county, Liaoyang, Liaoning, China
LE-3	*L. erythrorhizon*	Root	Benxi county, Benxi, Liaoning, China
LE-4	*L. erythrorhizon*	Root	Huanren county, Benxi, Liaoning, China
LE-5	*L. erythrorhizon*	Root	Nanfen district, Benxi, Liaoning, China
LE-6	*L. erythrorhizon*	Root	Changhai county, Dalian, Liaoning, China
LE-7	*L. erythrorhizon*	Root	Wafangdian county, Dalian, Liaoning, China
LE-8	*L. erythrorhizon*	Root	Kuandian county, Dandong, Liaoning, China
LE-9	*L. erythrorhizon*	Root	Fengcheng county, Dandong, Liaoning, China
LE-10	*L. erythrorhizon*	Root	Tieling county, Tieling, Liaoning, China
LE-11	*L. erythrorhizon*	Root	Changtu county, Tieling, Liaoning, China
LE-12	*L. erythrorhizon*	Root	Xifeng county, Tieling, Liaoning, China
LE-13	*L. erythrorhizon*	Root	Youyan district, Anshan, Liaoning, China
LE-14	*L. erythrorhizon*	Root	Datong district, Daqing, Heilongjiang, China
LE-15	*L. erythrorhizon*	Root	Lindian county, Daqing, Heilongjiang, China

### 2.2 Instrumentations and analytical conditions

All experiments were carried out on a Waterson H-CLASS ultra-high-performance liquid chromatography Xevo TQ-S triple quadrupole tandem mass spectrometer. The chromatographic column was an HSS T3 column (2.1 mm × 100 mm, 1.8 μm), and the column temperature was 40°C. The mobile phase comprised acetonitrile containing 0.1% formic acid (A) and water containing 0.1% formic acid (B). The gradient duration program was 3%–5% A (0–8 min), 5%–15% A (8–18 min), 15%–20% A (18–22 min), 20%–50% A (22–26 min), 50%–3% A (26–27 min), and 3% A (27–30 min). The flow rate was 0.3 mL/min, and the injection volume was 1 μL.

A Xevo TQ-S triple quadrupole tandem mass spectrometer equipped with an electrospray ionization (ESI) source (MA, United States) was employed to determine the contents of eight PAs in all samples. The mass spectra were obtained in the positive mode, and the parameters were as follows: dry gas (N_2_) flow rate, 10 L/min at the temperature 450°C; sheath gas flow, 8 L/min with a heater at 350°C; nebulizer pressure, 45 psi; capillary voltage, 4000 V for ESI, 500 V charging; and dwell time, 20 ms for each ion pair. In addition, PAs were determined in multiple reaction monitoring (MRM) acquisition modes. Other details are shown in Table 2.

**TABLE 2:** Selected MRM transitions and compound parameters for the 8 PAs.

Compounds	Retention time (min)	Precursor ion (*m/z*)	Quantifier ion (*m/z*)	Qualifier ion (*m/z*)	Cone voltage (V)	Collision voltage (V) (quant/qual)
Im	7.96	300.2	94.0	138.0	14	24/16
Ly	8.32	300.2	94.1	138.1	18	24/16
ImN	9.70	316.2	172.0	94.0	36	30/34
LyN	10.03	316.2	172.1	94.0	52	26/36
AcLy	14.12	342.2	120.0	180.1	12	24/16
AcLyN	14.45	358.2	214.1	180.1	16	26/28
EmN	18.92	414.3	254.2	352.	78	30/22
Em	19.18	398.3	120.0	220.1	70	20/16

### 2.3 Standard solutions and calibration curves

Eight PA reference standards were dissolved in 1.00 μg/mL stock solutions using 20% ACN aqueous solution. The mixed reference solution (20 ng/mL) was obtained by diluting the stock solutions with the same solvent. The calibration curves of mixed standards were obtained at a series of concentrations (1.0, 2.0, 5.0, 10.0, 20.0, 50.0, and 100 ng/mL) by diluting the stock solution.

### 2.4 Sample preparation and quality control samples

The dried powder (1.0 g) of the sample was weighed and placed in a centrifuge tube, and 40 mL of 0.1% formic acid aqueous solution was added, after which the samples were extracted by ultrasound (40 kHz, 300 W) for 20 min. Then, the extracting solution was centrifuged (6,000 rpm, 5 min), and 10 mL of the supernatant was subject to a cation-exchange solid-phase extraction (SPE) cartridge. The cartridge was subsequently eluted with 10 mL of methanol (the extract was removed). The alkaloid fractions were eluted with 10 mL of methanol–25% ammonia (3:1, *v*/*v*) and then evaporated to dryness under reduced pressure. The residue was dissolved in 100 mL of 20% ACN aqueous solution and filtered through a 0.22-μm membrane. Some batches of samples with high concentration were diluted with 20% acetonitrile ten times before injection.

The quality control (QC) samples were prepared by mixing the first batch of the three medicinal Zicao (sample No. AE-1, AG-1, and LE-1) in the same proportion to validate the precision, repeatability, and accuracy.

### 2.5 Method validation

The established UHPLC–MS/MS method was used for complete methodological validation, including linearity, the lower limit of detection (LLOD), the lower limit of quantification (LLOQ), intra-day and inter-day precision, stability, repeatability, accuracy, and matrix effect.

The sensitivity was determined by diluting the reference solution and injecting the sample. The lowest concentration when the signal-to-noise ratio was 10 was the LLOQ, and the lowest concentration when the signal-to-noise ratio was 3 was LLOD. The calibration curves were plotted by the concentration of the standard as the *x*-axis and the corresponding average peak area for two consecutive injections of each concentration as the *y*-axis. The inter-day precision was calculated by injecting the mixed reference solution six consecutive times, and the intra-day precision was calculated by injecting the mixed reference solution twice a day for three consecutive days. The stability was measured by calculating the relative standard deviation (RSD) of the peak area of a QC sample after it was placed at controlled room temperature (20°C–25°C) for 0, 2, 4, 8, 12, and 24 h. The repeatability was determined by calculating the RSD of the content of six QC samples prepared in parallel. The recovery test was used to evaluate the accuracy of the method. It was carried out by adding the known amount of eight standards at the same level as the known amount to a certain amount (0.5 g) of QC samples. Then, the spiked sample was extracted and analyzed by the method above, and the average recovery rate was calculated using the following formula: recovery rate (%) = (observed amount − original amount)/spiked amount × 100%.

The matrix effects (MEs) were calculated as the ratios of the slopes of the calibration curves prepared by the sample solution as the matrix to the slopes of the calibration curves prepared by the blank solution.

A series of standard solutions with different concentrations were prepared using the sample solutions of the first batch (sample No. AE-1, AG-1, and LE-1) of the three medicinal Zicao as the matrix. The ME was calculated using the following formula: ME (%) = *k*_matrix_/*k*_solvent_ × 100%, where *k*_matrix_ is the slope of the matrix-matched calibration curve, and *k*_solvent_ is the slope of the pure solvent calibration curve. In general, if the ME was 80%–120%, it could be ignored; if the ME was > 120%, the signal was considered to be enhanced; however, if it was < 80%, the signal was considered to be suppressed.

### 2.6 Identification and quantification

The target peak was determined by comparing UHPLC retention time and mass/charge ratio (*m*/*z*) with its standard. The linear calibration plots of peak areas and concentrations were used for quantitative analysis.

### 2.7 Estimation of the daily intake of three medicinal Zicao based on interim REP

The interim REP factors were developed by calculating the estimated daily intake (EDI) value (μg/kg bw/day) to examine the carcinogenic health risk of PAs in the three medicinal Zicao (Merz and Schrenk, 2016). PAs could be grouped based on their toxicity, provided they acted by a common mode of action. Several abundant PAs suggested a factor of 1.0 for cyclic di-esters and open-chain di-esters with 7*S* configuration, 0.3 for mono-esters with 7*S* configuration, 0.1 for open-chain di-esters with 7*R* configuration, and 0.01 for mono-esters with 7*R* configuration (Schrenk et al, 2020). Additionally, the unique exposure characteristics of traditional Chinese medicine (TCM) were considered by calculating the EDI (μg/kg bw/day) as follows:

EDI=EF×Ed×IR×C×REF/W×AT

where EDI is the estimated daily intake of PAs in the three medicinal Zicao; EF represents exposure frequency, which was 90 days/year; Ed is exposure duration, which was 20 years according to a previous study by our team; and IR is the daily intake rate of the three medicinal Zicao. Based on Pharmacopoeia of the People’s Republic of China guidelines, the average IR is 10 g/day; C is the PA content in the three medicinal Zicao (μg/g); REF is the interim REP factor; W represents the mean body mass, which is 70 kg for adults according to the EFSA; and AT is the average exposure time to the three medicinal Zicao, which was 365 days/year × 70 years.

Compared with oral administration, the absorption rate of Zicao for external use was greatly reduced. In their study, Plaza et al. reported that the skin absorption rate of 7*R* open-chain di-ester PAs and 7*R* monoester PAs in humans was 7.25% and 6.98%, respectively. In this study, PAs 1–4 were 7R monoesters, and 5–8 were 7*R* open-chained di-esters. For external use, the EDI value was multiplied by the absorptivity.

### 2.8 MOE assessment

The MOE modeling strategy determined the risk assessment. EFSA considers 237 μg/kg bw/dayas the reference point for riddelliine, which was used as a starting point for assessing the carcinogenic risk of PAs by means of an updated benchmark dose strategy. The present study obtained MOE values for various PAs by dividing the BMDL_10_ of 237 μg/kg bw/day for riddelliine by the EDI. According to the MOE value, herbs were divided into four levels, i.e., high risk (<100), medium risk (100–1,000), low risk (1,000–10,000), and no risk (>10,000) (Knutsen et al., 2017; Wang et al., 2021).

## 3 Results and discussion

### 3.1 Method development and optimization

Different mobile phases were compared to obtain the best separation and ionization efficiency: 1) acetonitrile-water containing 0.1% formic acid, 2) acetonitrile-water containing 0.1% ammonium formate, and 3) acetonitrile-water containing 0.1% formic acid and 5 mmol/L ammonium formate. Multiple injections showed that eight PAs’ sensitivity greatly improved when formic acid and ammonium formate were added to the mobile phase. Im, Ly, and their corresponding nitrogen oxides were diastereomers with the same ionic transition; hence they needed to be separated by chromatography. Also, the mobile phase proportion and flow rate were adjusted to achieve good separation. The best mass spectrum parameters, cone voltage, and collision voltage were obtained by comparing the ionization intensity of the reference solution.

### 3.2 Optimization of sample preparation

The extraction efficiency of the sample was measured using the content of compounds in the QC sample. The extraction efficiencies of the following three solvents were compared: 1) 0.1% formic acid water, 2) 0.05 mol/L sulfuric acid aqueous solution, and 3) water. The extraction rate of alkaloids from acidified water was significantly higher than that from pure water, while formic acid water and sulfuric acid water had similar extraction rates. Considering that sulfuric acid was more dangerous, formic acid water was selected as the extraction solvent in this experiment. Therefore, the cation-exchange SPE column was suitable for the purification of PAs. The purification efficiency of different brands of SPE columns was compared with the recovery rates of the reference standards. The recovery rates of three brands of SPE columns (Oasis MCX 6 mL/150 mg, 60 μm, Waters; SHIMSEN StyraSCX 6 mL/500 mg, Shimadzu; Hypersep SCX 6 mL/500 mg, Thermo Scientific) could reach 80%–120%. Oasis MCX was slightly better than the other two brands. Therefore, the Oasis SPE column was selected for the experiment.

### 3.3 Method validation

#### 3.3.1 Selectivity

**FIGURE 2:**
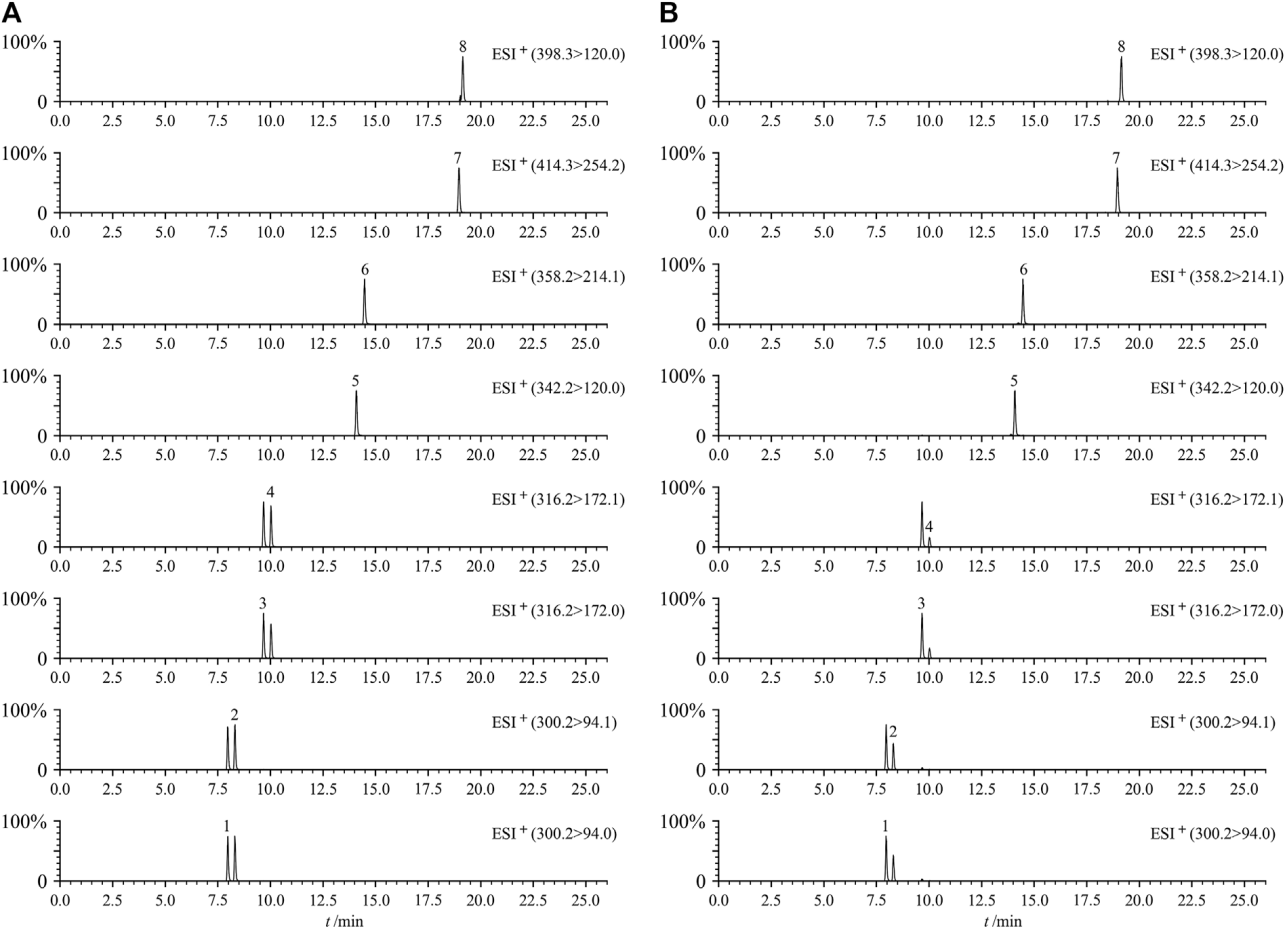
Typical UHPLC–MS/MS chromatograms (MRM) of eight PAs of mixed standard solution **(A)** and QC sample **(B)**. Intermedine (1), lycopsamine (2), intermedine *N*-oxide (3), lycopsamine *N*-oxide (4), 7-acetyllycopsamine (5), 7-acetyllycopsamine *N*-oxide (6), echimidine *N*-oxide (7), and echimidine (8).

Figure 2 shows the MRM diagram of the eight cross-reference substances to be tested and the MRM representative chromatograms of QC samples. The eight components to be tested could achieve baseline separation, and no potential interfering components were observed in the spectrum. The results showed that the method had good selectivity in determining these eight PAs in the three medicinal Zicao.

#### 3.3.2 Calibration curve, LLODs, and LLOQs

As shown in Table 3, the standard curves of all analytes had a good linear relationship within the tested concentration range, and the correlation coefficients were > 0.999. LLODs and LLOQs were in the range of 0.095–0.20 ng/mL and 0.28–0.51 ng/mL, respectively, indicating that the method had high sensitivity.

**TABLE 3:** Calibration curves, LLOD and LLOQ of the 8 PAs.

Compounds	Calibration curves	*r*	Linear range/(ng·mL^−1^)	LLOD/(ng·mL^−1^)	LLOQ/(ng·mL^−1^)
Im	*Y* = 12102*X*-1258	0.999 2	1.004–100.4	0.10	0.30
Ly	*Y* = 11204*X*-854	0.999 1	0.948–94.8	0.095	0.28
ImN	*Y* = 8750*X*-1085	0.999 3	0.982–98.2	0.20	0.49
LyN	*Y* = 7963*X*-752	0.999 1	1.022–102.2	0.20	0.51
AcLy	*Y* = 2635*X*-2101	0.999 2	1.016–101.6	0.19	0.51
AcLyN	*Y* = 6952*X*-791	0.999 4	0.966–96.6	0.19	0.48
EmN	*Y* = 5401*X*-129	0.999 2	0.988–98.8	0.20	0.49
Em	*Y* = 4985*X*-632	0.999 4	0.994–99.4	0.20	0.50

#### 3.3.3 Precision, repeatability, and stability

RSD% expressed the results of precision, repeatability, and stability. The results are shown in Table 4. The RSD values of intra-day and inter-day precision, repeatability, and stability were all < 4.0%. These results met the allowable range of Food and Drug Administration (FDA) standard rules.

**TABLE 4:** Precision, repeatability, stability and recovery results of the 8 PAs.

Compounds	Precision (%, *n* = 6)		Repeatability (%, *n* = 6)	Stability (%, *n* = 6)	Recovery (%, *n* = 3)	
	Intra-day	Inter-day			Mean	RSD
Im	1.75	2.32	2.25	2.25	91.25	1.91
Ly	2.14	2.62	2.58	2.36	93.62	2.46
ImN	2.38	3.41	3.21	3.29	89.26	1.91
LyN	2.45	3.31	3.54	3.14	92.88	3.24
AcLy	3.33	3.74	3.71	3.68	87.63	3.35
AcLyN	3.12	3.58	3.63	3.85	89.14	2.75
EmN	2.23	3.34	3.56	2.36	90.74	3.58
Em	2.65	2.95	3.88	2.25	93.18	3.47

#### 3.3.4 Recovery and ME

The recovery results are shown in Table 4. The recoveries of eight PAs were between 87.63% and 93.62%, and the RSD was < 4.0%. The MEs of the three medicinal Zicao AE, AG, and LE was 85.23%–112.52%, 86.32%–113.25%, and 84.14%–112.38%, respectively (Table 5). No significant ME was found in the established assay.

**TABLE 5:** MEs (%) of 8 PAs in three Zicao by the established UPLC-MS/MS.

Compounds	MEs (%)		
	AE	AG	LE
Im	85.23	89.63	84.14
Ly	87.42	92.85	87.12
ImN	91.62	86.32	105.32
LyN	88.27	89.26	112.38
AcLy	105.36	113.25	91.26
AcLyN	108.92	110.29	87.14
EmN	112.52	107.14	89.52
Em	109.63	106.32	91.25

### 3.4 Analysis of PAs in the three medicinal Zicao

The established UHPLC–MS/MS method was used to determine the content of eight PAs in three medicinal Zicao, revealing that the types and content of PAs in the three medicinal Zicao were quite different (Table 6). Im and ImN were common components in the eight PAs tested. AcLy and AcLyN were not detected in AE; Em and EmN were not detected in AG; Ly, LyN, AcLy, and AcLyN were not detected in LE. The total content of 8 PAs in 11 batches of AG was 341.56–519.51 μg/g, with an average of 448.99 μg/g; the content in 15 batches of LE was 71.16–515.73 μg/g, with an average of 271.62 μg/g; the content in 11 batches of AE was 23.35–207.13 μg/g, with an average of 89.47 μg/g. LyN had the highest content in AE (6.93–111.75 μg/g). The highest one in AG was ImN (185.63–319.85 μg/g), while EmN in LE was the most abundant PA with a content of 28.47–238.79 μg/g.

**TABLE 6:** The results of sample determination (μg·g^−1^).

Codes	Im	Ly	ImN	LyN	AcLy	AcLyN	EmN	Em	Total
AE-1	0.81	4.77	16.85	57.23	-	-	2.76	3.22	85.64
AE-2	0.52	6.01	14.28	87.74	-	-	2.31	1.04	111.90
AE-3	0.42	2.45	12.41	47.15	-	-	2.71	1.38	66.52
AE-4	0.59	3.06	15.48	44.21	-	-	22.74	4.52	90.60
AE-5	0.44	0.55	13.71	11.29	-	-	33.25	10.21	69.45
AE-6	1.29	4.92	50.83	107.63	-	-	5.45	1.55	171.67
AE-7	1.77	6.82	65.15	111.75	-	-	15.32	6.32	207.13
AE-8	0.47	0.53	10.93	8.59	-	-	5.63	1.03	27.18
AE-9	0.45	0.65	6.86	6.93	-	-	4.21	4.25	23.35
AE-10	0.43	1.68	10.35	22.32	-	-	2.12	4.32	41.22
AE-11	0.72	3.14	21.69	50.48	-	-	9.65	3.78	89.47
AG-1	49.26	30.32	316.71	51.52	20.44	2.69	-	-	470.94
AG-2	49.82	20.32	319.85	78.65	45.66	5.21	-	-	519.51
AG-3	56.32	25.81	258.66	69.32	21.54	2.54	-	-	434.19
AG-4	52.41	35.21	254.18	48.74	18.45	3.52	-	-	412.51
AG-5	64.14	24.26	185.63	32.45	30.52	4.56	-	-	341.56
AG-6	32.02	46.25	306.32	85.24	28.62	3.35	-	-	501.80
AG-7	35.63	47.21	226.42	40.21	38.95	5.85	-	-	394.27
AG-8	45.41	54.74	304.21	63.52	22.54	10.08	-	-	500.50
AG-9	54.12	36.45	246.52	53.21	28.96	4.25	-	-	423.51
AG-10	39.24	11.44	329.11	75.62	34.21	1.45	-	-	491.07
AG-11	47.84	33.20	274.76	59.85	28.99	4.35	-	-	448.99
LE-1	10.71	-	86.75	-	-	-	36.92	27.07	161.45
LE-2	9.21	-	223.52	-	-	-	238.79	44.21	515.73
LE-3	6.31	-	95.62	-	-	-	195.32	10.82	308.07
LE-4	8.21	-	105.32	-	-	-	85.47	16.38	215.38
LE-5	2.52	-	150.65	-	-	-	92.35	24.12	269.64
LE-6	9.42	-	102.34	-	-	-	114.85	74.36	300.97
LE-7	2.06	-	63.58	-	-	-	169.74	34.18	269.56
LE-8	3.36	-	92.52	-	-	-	185.47	19.67	301.02
LE-9	9.95	-	175.14	-	-	-	96.68	25.35	307.12
LE-10	4.61	-	77.85	-	-	-	174.24	41.52	298.22
LE-11	6.25	-	121.74	-	-	-	156.84	18.28	303.11
LE-12	9.74	-	76.96	-	-	-	125.65	22.35	234.70
LE-13	5.81	-	48.65	-	-	-	174.16	17.87	246.49
LE-14	2.28	-	36.96	-	-	-	28.47	3.45	71.16
LE-15	6.46	-	104.11	-	-	-	133.93	27.12	271.62

-: not detected.

### 3.5 Health risk assessment

As comprehensive data on the toxicity of all congeners cannot be generated due to a large number of PAs, congener-specific data on their relative toxicity are needed. The concept of REP factors has been established because the individual potencies of members of such groups, termed congeners, can be extremely different, even spanning several orders of magnitude. This approach is based on the concept that all carcinogenic PAs share a common mode of action. It describes each congener’s relative (toxic) potency compared with the most toxic congener(s). The latter (such as riddelliine) is assigned a REP factor of 1.0 describing its toxicity as 100%. All other congeners had REP ≤ 1.0. In the present study, the REP for Im, Ly, ImN, and LyN was 0.01, and the REP for AcLy, AcLyN, EmN, and Em was 0.1. Multiplying the amount of a congener by its REP factor and summing up these products resulted in an overall sum of equivalents in the Zicao sample, which was then taken as a suitable parameter quantitatively describing the toxicity of Zicao. Similar well-established approaches were suggested for polychlorinated dioxins, furans, phototoxic furocoumarins, and synthetic glucocorticoids (Van den Berg et al., 2006; Raquet and Schrenk, 2014; Suzuki et al., 2015).

The present study considered the REP factor approach in a MOE-based PA risk assessment in Chinese herbal medicines (CHMs). The risk was assessed for the three medicinal Zicao by considering the relative toxicity of PAs with different chemical structures. If the worst-case hypothesis was used, which assumes all PAs as equally potent as the most toxic PAs, such as riddelliine (JECFA, 2015), it could result in an overestimation of the risks among patients and lead to misleading inappropriate restrictions on using valuable medicines. In addition, given the differences between TCMs and food, we obtained the exposure frequency and duration data based on our questionnaire and built a risk assessment model that could evaluate the risks of PAs based on fundamental structural considerations. Thus, the present study provided novel insights into the risk assessment of PAs to obtain a more realistic and scientific conclusion. This study also calculated the MOE values using conventional MOE methods. The MOE value for the three medicinal Zicao was 114–1,015, 47–69, and 51–333, respectively, and the corresponding risks were high, which overestimated the toxicity risk of Zicao. Combined with the REP factor, the MOE value was 5,146–23,896, 2,425–3,884, and 925–6,612, respectively. Except for one batch of LE, the rest were in the low-risk range, which was consistent with the toxicity reports of these herbs. The average total contents of PAs in AG were higher than AE and LE, but ImN in AG was the component with the highest content. ImN is 7*R* open-chain mono-ester PA, and REF was only 0.01. However, both Em and EmN in LE had high contents. Em and EmN are 7*R* open-chain di-ester PAs, and their REF is 0.1. Therefore, the MOE values for LE combined with REF were lower than the other two. Because the REFs of PAs 1–4 were one-tenth those of 5–8, the PA with higher content in 5-8 had greater contribution to risk and were worthy of attention. EmN is the largest contributor to risk for AE and LE, while AcLy for AG. For external use, the MOE values were > 10,000 due to the low transdermal absorption rate, posing no health risk. The EDI and MOE value of the three medicinal Zicao by conventional methods and the results of oral and external administration combined with REP factor are listed in Table 7.

**TABLE 7:** EXPand MOE values of three Zicao in three cases.

Codes	EXP^a^	EXP^b^	EXP^c^	MOE^a^	MOE^b^	MOE^c^
AE-1	0.856	0.0139	0.00099	277	16,994	239,986
AE-2	1.119	0.0142	0.00100	212	16,684	237,313
AE-3	0.665	0.0103	0.00073	356	22,936	324,253
AE-4	0.906	0.0336	0.00242	262	7055	98,066
AE-5	0.695	0.0461	0.00333	341	5,146	71,151
AE-6	1.717	0.0235	0.00165	138	10,099	143,483
AE-7	2.071	0.0402	0.00286	114	5,897	82,955
AE-8	0.272	0.0087	0.00063	872	27,204	379,196
AE-9	0.234	0.0099	0.00072	1,015	23,821	330,744
AE-10	0.412	0.0099	0.00071	575	23,896	334,489
AE-11	0.895	0.0210	0.00150	265	11,268	157,783
AG-1	4.709	0.0679	0.00476	50	3,490	49,765
AG-2	5.195	0.0977	0.00692	46	2,425	34,263
AG-3	4.342	0.0651	0.00457	55	3,641	51,843
AG-4	4.125	0.0610	0.00428	57	3,884	55,327
AG-5	3.416	0.0657	0.00465	69	3,606	50,914
AG-6	5.018	0.0790	0.00555	47	3,002	42,665
AG-7	3.943	0.0797	0.00566	60	2,972	41,904
AG-8	5.005	0.0794	0.00559	47	2,985	42,407
AG-9	4.235	0.0722	0.00510	56	3,281	46,499
AG-10	4.911	0.0812	0.00572	48	2,919	41,411
AG-11	4.490	0.0749	0.00528	53	3,164	44,878
LE-1	1.615	0.074	0.0053	147	3,214	44,626
LE-2	4.657	0.256	0.0185	51	925	12,814
LE-3	3.081	0.216	0.0156	77	1,096	15,146
LE-4	2.154	0.113	0.0082	110	2094	29,022
LE-5	2.696	0.132	0.0095	88	1798	24,949
LE-6	3.010	0.200	0.0145	79	1,183	16,359
LE-7	2.696	0.210	0.0152	88	1,126	15,555
LE-8	3.010	0.215	0.0155	79	1,104	15,258
LE-9	3.071	0.141	0.0101	77	1,686	23,413
LE-10	2.982	0.224	0.0162	79	1,058	14,620
LE-11	3.031	0.188	0.0136	78	1,261	17,455
LE-12	2.347	0.157	0.0113	101	1,513	20,923
LE-13	2.465	0.197	0.0143	96	1,200	16,576
LE-14	0.712	0.036	0.0026	333	6,612	91,698
LE-15	2.716	0.172	0.0124	87	1,377	19,055

^a^ Calculated by routine methods.

^b^ Calculated by relative potency factor methods.

^c^ Calculated by relative potency factor methods in case of transdermal absorption as an external drug.

## 4 Conclusion

The present study established a UHPLC–MS/MS method for simultaneously determining and validating eight PAs in the three medicinal Zicao. Significant differences were found in the types and contents of PAs among the three medicinal Zicao. The novelty of this study is a comprehensive analysis of PAs in Zicao and reporting LyN from AE, six compounds (Im, Ly, ImN, LyN, AcLy, and AcLyN) from AG, and three PAs (ImN, Em, and EmN) from LE. The risk of PAs in Zicao was assessed by conventional MOE combined with the REP factor method under oral and external usages. The reported results might be of great significance for the rational use of Zicao. In addition, the risk assessment of PAs by this method also has a reference value for other TCMs.

It should be acknowledged that this study has also some limitations. Although we have made every effort to purchase all PAs on the market to screen and determine the PA of Zicao, there are still some PAs of Zicao whose reference standards were not available and have not yet been determined. Therefore, the total PA content in Zicao may be higher than the eight components determined in this study. According to the total content of these eight PAs, the risk and toxicity of Zicao PAs may be underestimated. It is still a challenge to determine the content of components without reference standards in traditional Chinese medicine. In the future, sufficient pure compounds may be obtained through phytochemistry or organic synthesis to comprehensively determine PAs in Zicao.

## Data Availability

The original contributions presented in the study are included in the article/Supplementary Material, further inquiries can be directed to the corresponding authors.

## Abbreviations

AcLy, 7-acetyllycopsamine; AcLyN, 7-acetyllycopsamine *N*-oxide; ACN, acetonitrile; AE, *Arnebia euchroma*; AG *Arnebia guttata*; BMDL_10_, benchmark dose lower confidence limit for 10%; ESI, electrospray ionization; CHM, Chinese Herbal Medicines; Em, echimidine; EmN, echimidine *N*-oxide; HILIC-MS, hydrophilic interaction liquid chromatography–mass spectrometry; Im, intermedine; ImN, intermedine *N*-oxide; LE, *Lithospermum erythrorhizon*; LLOD, lower limit of detection; LLOQ, lower limit of quantification; Ly, lycopsamine; LyN, lycopsamine *N*-oxide; ME, matrix effect; MOE, margin of exposure; MRM, multiple reaction monitoring; PA, pyrrolizidine alkaloid; QC, quality control; REP, relative potency; RSD, relative standard deviation; SPE, solid-phase extraction; TCM, traditional Chinese medicine; UHPLC-QTOF-MS, ultra-high-performance liquid chromatography–quadrupole time-of-flight–mass spectrometry; UHPLC–MS/MS, ultra-high-performance liquid chromatography coupled with tandem mass spectrometry.
